# The Effect of *In Vitro* Cultivation on the Transcriptome of Adult *Brugia malayi*

**DOI:** 10.1371/journal.pntd.0004311

**Published:** 2016-01-04

**Authors:** Cristina Ballesteros, Lucienne Tritten, Maeghan O’Neill, Erica Burkman, Weam I. Zaky, Jianguo Xia, Andrew Moorhead, Steven A. Williams, Timothy G. Geary

**Affiliations:** 1 Institute of Parasitology, Centre for Host-Parasite Interactions, McGill University, Sainte-Anne-de-Bellevue, Quebec, Canada; 2 Department of Infectious Diseases, College of Veterinary Medicine, University of Georgia, Athens, Georgia, United States of America; 3 Filariasis Research Reagent Resource Center, Northampton, Massachusetts, United States of America; 4 Department of Biological Sciences, Smith College, Northampton, Massachusetts, United States of America; New York University, UNITED STATES

## Abstract

**Background:**

Filarial nematodes cause serious and debilitating infections in human populations of tropical countries, contributing to an entrenched cycle of poverty. Only one human filarial parasite, *Brugia malayi*, can be maintained in rodents in the laboratory setting. It has been a widely used model organism in experiments that employ culture systems, the impact of which on the worms is unknown.

**Methodology/Principal Findings:**

Using Illumina RNA sequencing, we characterized changes in gene expression upon *in vitro* maintenance of adult *B*. *malayi* female worms at four time points: immediately upon removal from the host, immediately after receipt following shipment, and after 48 h and 5 days in liquid culture media. The dramatic environmental change and the 24 h time lapse between removal from the host and establishment in culture caused a globally dysregulated gene expression profile. We found a maximum of 562 differentially expressed genes based on pairwise comparison between time points. After an initial shock upon removal from the host and shipping, a few stress fingerprints remained after 48 h in culture and until the experiment was stopped. This was best illustrated by a strong and persistent up-regulation of several genes encoding cuticle collagens, as well as serpins.

**Conclusions/Significance:**

These findings suggest that *B*. *malayi* can be maintained in culture as a valid system for pharmacological and biological studies, at least for several days after removal from the host and adaptation to the new environment. However, genes encoding several stress indicators remained dysregulated until the experiment was stopped.

## Author Summary

Infections with filarial worms cause serious physical impairment and affect tens of millions of people in tropical and subtropical countries. To better understand the biology and pharmacology of these parasites, *Brugia malayi* is often used as a model. This parasite can be maintained in the laboratory in Mongolian jirds, enabling researchers to test drugs *in vivo* and *in vitro*, among other studies. The effects of removing worms from their hosts and culturing them may affect many aspects of their physiology, including response to drugs, but the extent to which the worms undergo changes during culture has remained unknown. Using deep RNA sequencing and bioinformatics tools, we examined the global transcriptomic profile of *B*. *malayi* females at four different time points over 5 days in culture. Focusing on genes that are differentially expressed at various time points, we observed a general perturbation of the expression profile between dissection from the host and receipt after shipment. The expression of several genes remained changed at the end of the experiment, after 5 days under controlled conditions; in particular, genes encoding cuticle collagens were prominently represented and strongly overexpressed.

## Introduction

Lymphatic filariasis (LF) is a neglected tropical disease caused by three filarial nematodes: *Wuchereria bancrofti*, *Brugia malayi*, and *Brugia timori*, which are transmitted by several species of mosquitoes [1]. LF is presently endemic in 60 countries, mainly in subtropical and tropical regions of the world. It is estimated that over 120 million people are currently infected and up to 800 million people are at risk [2]. Chronic LF can lead to severe disabilities due to clinical manifestations such as chronic lymphoedema (elephantiasis) and hydrocoele in men, and those affected are often plagued by social stigma and adverse economic consequences [3].

In 1994, the UNDP/World Bank/World Health Organization Special Programme for Research and Training in Tropical Diseases (TDR) initiated The Filarial Genome Project (FGP); *B*. *malayi* was chosen as a model organism due to the availability of all life cycle stages for the construction of cDNA libraries [4]. In 2007, the nuclear and mitochondrial genomes of this parasite were sequenced, as well as the genome of its bacterial endosymbiont *Wolbachia* [5]. Access to genomic data is key to advancing our understanding of parasitic nematodes and developing new ways to control and eliminate diseases caused by them.

*In vitro* studies are vital to the advancement of filariasis research. A weakness of *in vitro* culture systems for all pathogens, especially metazoans such as helminths, is that they do not accurately replicate the physiological conditions at the infection site in a host, as evidenced by the inability to maintain prolonged viability of adult stages. Hence, culture studies provide results that are of uncertain relevance for the biology of the parasite *in situ*.

Organisms have the ability to sense and adapt to environmental changes (short or long term) to maintain homeostasis [6]. Alteration of gene expression plays an important role in adaptation, with extensive regulation at the transcriptional and post-transcriptional levels. Changes in environmental factors such as temperature, humidity, water, light intensity, supply of nutrients, and interactions with other organisms (i.e., infection or mechanical damage) can lead to stress and altered gene expression patterns [7]. Subsequently, changes in gene expression can occur which are not usually observed in the unstressed organism. An example is the production of heat shock proteins as a specific response to elevated temperatures, and modification of basic metabolism as a non-specific response [8].

The goal of this study was to evaluate changes in gene expression over time upon *in vitro* maintenance of adult *B*. *malayi* female worms in culture as an index of adaptation to removal from the host. We examined the worm’s global transcriptome by Illumina sequencing technology, a method shown to be highly replicable for identifying differentially expressed genes [9], from the time the parasites were extracted from jirds in Georgia (USA), shipped to Montreal (Canada), and after maintenance for up to 5 days in culture under controlled conditions. A number of *in vitro* drug testing studies have relied on worms shipped by the NIH-NIAID Filariasis Research Reagent Resource Center (FR3) at the University of Georgia [10, 11] with timing and conditions similar to those employed in the present work.

## Materials and Methods

### Ethical statement

All animal procedures were approved by the University of Georgia Institutional Animal Care and Use Committee and complied with U.S. Department of Agriculture regulations (USDA Assurance No. A3437-01).

### Worms and study design

**Fig 1:**
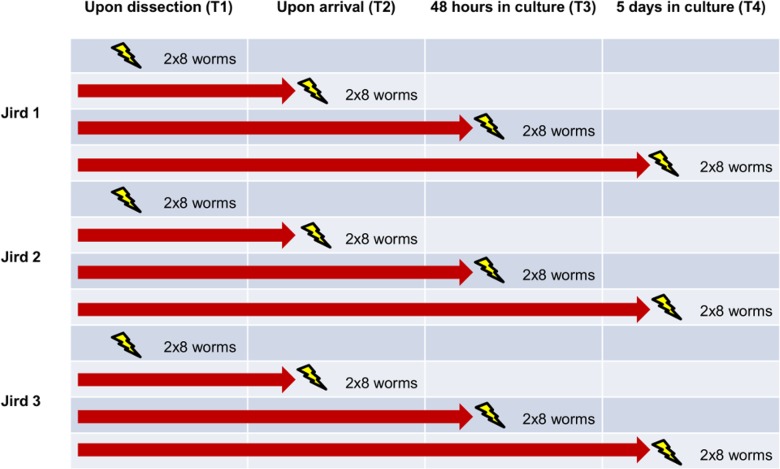
Study design. At each time point, two groups of 8 worms from each jird were washed, flash-frozen and used for RNA extraction.

Adult male jirds (*Meriones unguiculatus*) were injected subcutaneously with ≈400 *B*. *malayi* infective third-stage larvae (L_3_). After a minimum of 90 days post-infection (ranging from 3 to 6 months), jirds were euthanized by exposure to CO_2_ and adult worms were collected from the peritoneal cavity via lavage. Using 3 jirds in total, female worms recovered upon necropsy from an individual jird were assigned to 8 groups (4 time points, 2 technical replicates) of 8 worms without randomization, to assess transcriptomic variability attributable to host of origin (Fig 1). Worms selected for the first group (T1) were thoroughly washed in sterile PBS and flash-frozen in liquid N_2_ before being shipped on dry ice to McGill University. The remaining groups of 8 were shipped overnight in separate 15 ml tubes containing RPMI-1640 (Lonza, Walkersville MD) and 1% gentamycin (Gentamycin solution, 10 mg/ml, Sigma Aldrich, St. Louis, MO) via FedEx from Georgia to Montreal.

Upon arrival at McGill, two separate groups of 8 worms from each jird were washed 3 times in sterile PBS and used for RNA extraction (T2). The remaining groups were incubated 1 worm per well of a 12-well plate (Costar) containing 6 ml RPMI 1640 (Sigma-Aldrich, St. Louis MO) supplemented with 10% v/v heat-inactivated fetal bovine serum (Sigma-Aldrich, F1051), 5% penicillin/streptomycin (Sigma–Aldrich) and 2% gentamycin (Gibco, 15750–060) for either 48 h (T3) or 5 days (T4) at 37°C in 5% CO_2_. On a daily basis, 3 ml of culture medium in each well was replaced with fresh medium.

### RNA extraction

Surfaces were washed with RNase AWAY (Molecular BioProducts) and all dilutions were prepared with UltraPure distilled water (Invitrogen, Life Technologies, Burlington, ON). Washed live worms were pooled in 1.5-ml tubes; immotile worms were excluded. RNA was extracted from 5 to 8 worms per group. For both live and already frozen worms (group T1), 125 μl 0.1X nuclease-free Tris-EDTA (TE) buffer, pH 8.0 (Ambion, Life Technologies, Burlington, ON) and 375 μl Trizol LS reagent (Ambion) were added to each tube on ice. Three cycles of flash-freezing in liquid N_2_ and crushing of worms in TE/Trizol LS with plastic pestles were performed to obtain homogeneous worm extracts. One hundred (100) μl chloroform was added to each tube and the samples were vortexed and incubated 3 min at room temperature. The mixtures were transferred to phase-lock gel heavy tubes (5 PRIME, Gaithersburg, MD) and centrifuged at 11,900 x *g* at 4°C for 15 min. The aqueous phase was transferred to fresh tubes and mixed with 250 μl ice-cold isopropanol. Tubes were centrifuged at 12,200 x *g* at 4°C for 30 min and left overnight at -20°C for RNA precipitation. Supernatants were discarded, pellets were washed twice with 80% EtOH and allowed to dry for several hours before resuspension in 50 μl 0.1X TE. A 10 min incubation at 55°C solubilized the pellet. Total RNA was purified and concentrated using an RNeasy Min-Elute Cleanup Kit (Qiagen, Valencia, CA). Samples were treated with DNase to remove contaminating DNA using an Ambion DNA-free Kit (Life Technologies, AM1906, Burlington, ON). The concentration and quality of the RNA for each sample was assessed by spectrophotometry (NanoDrop 1000, Wilmington, DE). RNA samples were shipped overnight on dry ice to the NIH-FR3 (Molecular Division) at Smith College (Northampton, MA) for cDNA library preparation and Illumina sequencing. RNA concentration was further verified using the Qubit RNA BR Assay Kit (Life Technologies, Q10210, Burlington, ON) and the integrity and purity was assessed on an Agilent 2100 Bioanalyzer (Santa Clara, CA).

### cDNA library preparation and Illumina sequencing

Messenger RNA (mRNA) was enriched with a NEBNext Poly (A) mRNA Magnetic Isolation Module (NEB, E7490, Ipswich, MA). Using the enriched mRNA as template, cDNA libraries were constructed using the NEBNext Ultra RNA Library Prep Kit Illumina (NEB, E7530, Ipswich, MA) and NEBNext Multiplex Oligos for Illumina (Index Primer 1–12) (NEB, E7600, Ipswich, MA) following the manufacturer’s instructions. To verify the quality, DNA concentration and product size of the cDNA libraries, a Qubit 2.0 Fluorometer (Life Technologies, Q32866), Qubit dsDNA BR assay kit (Life Technologies, Q32850), High Sensitivity DNA Analysis Kit (Agilent, 5067–4626) and Bioanalyzer were used. cDNA libraries were sequenced on an Illumina MiSeq Platform employing a 150 base pair single-end NGS setting. Data from MiSeq sequencing runs were uploaded and stored in BaseSpace (https://basespace.illumina.com) for data analysis.

### Data analysis

#### RNA sequencing analysis

Next generation sequencing unaligned raw data (fastq) files were downloaded from BaseSpace to the Mason-Galaxy platform (http://galaxy.iu.edu/ (Indiana University) [12, 13]. Files were groomed using Fastq Groomer (v 1.0.4) and quality control statistics were verified using FastQC: Reads QC (version 0.52). Quality and adapter trimming was performed using Fastq Quality Trimmer (v 1.0.0) and Trim Galore (v 0.2.8.1). Based on quality statistics, sequences were trimmed from both the 5’ and 3’ ends and adapter sequences removed. Tophat2 (v 0.6) was used for mapping gapped reads to the *B*. *malayi* reference genome (*B*. *malayi* V3 243 reference, ftp://ftp.wormbase.org/pub/wormbase/species/b_malayi/sequence/genomic/b_malayi.PRJNA10729.WS243.genomic.fa.gz). Picard alignment summary metrics were obtained from the alignment BAM files using SAM/BAM Alignment Summary Metrics (version 1.56.0) from Picard tools (http://broadinstitute.github.io/picard/) (Table 1). Differential expression analysis between time points was performed using edgeR (version 3.10.5) [14] in networkanalyst.ca [15, 16] after counting the number of reads per transcript using HTSeq-count (version 1.0.0) [17]. The union mode was used to handle reads overlapping more than one feature. All other parameters were kept at their default settings. Read counts for gene features were normalized in edgeR using the trimmed mean of M-values (TMM) method which corrects for different library sizes and reduces RNA compositional effect [18]. Tagwise (gene-specific) dispersion values were estimated by an empirical Bayes method based on weighted conditional maximum likelihood [19]. Pairwise differential expression testing between time points was performed using the exact T-test once negative binomial models were fitted and dispersion values estimated. Significance was assessed as having an experiment-wide false discovery rate (FDR) <0.01 (calculated using the Benjamini Hochberg method [20]). Genes which had a log_2_ fold-change value ≥ 1.0 or ≤-1.0 were further filtered and prioritized. Although we considered all significantly differentially expressed genes which had a FDR<0.01 biologically relevant, we applied an arbitrary fold-change cutoff to a level we considered interesting to limit the set of differentially expressed genes to a workable number for further analysis without applying too stringent a cutoff. A list of these genes for each comparison was imported into Microsoft Excel for further analysis. A Venn diagram was produced using Venny 2.0 (http://bioinfogp.cnb.csic.es/tools/venny/).

**Table 1:** Picard alignment summary metrics.

Time point	# Transcripts	Av. Total # Reads	Av. Total # Reads Mapped	Av. Total # High Quality Reads Mapped	% High Quality Reads Mapped
**T1 = upon dissection**	11485	1494658	1494570	1185542	79.32
**T2 = upon arrival**	11596	2352432	2352242	1702625	72.38
**T3 = 48 h**	11628	1290903	1290786	996403	77.19
**T4 = 5 days**	11456	1567087	1566944	1217739	77.71

Summary of the sequencing and mapping of the data to the *B*. *malayi* transcriptome from BAM files.

#### Bioinformatics analysis of the sequence data

From lists of filtered genes for each pairwise comparison between time points, the Wormbase Gene ID was used to retrieve the primary corresponding sequence name of the gene from Wormbase (http://www.wormbase.org/) and the UniProt accession number [21]. Available Gene Ontology (GO) terms [22] were obtained from the *B*. *malayi* gene table from nematode.net (v 4.0; http://nematode.net/NN3_frontpage.cgi) [23, 24], Gene Ontology (GO) term and KEGG/Panther pathway enrichment analysis was performed using KOBAS 2.0 [25]. As the free-living nematode *Caenorhabditis elegans* is exceptionally well-annotated and in the same phylum as *B*. *malayi*, we used the orthologs (Uniprot accession numbers) of differentially expressed genes found in this study for enrichment analysis, considering hits with a minimal E-value of 1*10^−20^.

To visualize the RNA sequencing data, customized heat maps were created using Network Analyst [15, 16] from a count table generated in HTSeq [17]. Genes were clustered according to Euclidean distance metrics. Specific heat maps were created to examine the changes in gene expression of genes found in Gene Ontology (GO) and pathway enrichment analysis and inter-jird variability.

#### Validation of quantitative gene expression

To validate Illumina sequencing results, 5 genes were chosen at random and gene expression was analyzed by quantitative polymerase-chain reaction (qPCR) for different pairwise comparisons. For each original RNA sample, 100 ng total RNA were reverse transcribed using the SuperScript VILO MasterMix (Invitrogen, #11755–050, Life Technologies, Burlington, ON) and diluted 5-fold for qPCR reactions. Real-time PCR was performed in triplicate using specific primers designed using Primer-BLAST (http://www.ncbi.nlm.nih.gov/tools/primer-blast/) for Bm8439, Bm9996, Bm7583, Bm1023, and Bm11095. Bm5699 (glyceraldehyde-3-phosphate dehydrogenase, GAPDH) was chosen as an endogenous control and normalizer, as its expression was found to be stable over time and across samples. S1 Table shows the selected candidate and reference genes used for the qPCR validation of the RNAseq data. Assays were carried out in 20 μl-reaction volumes containing 10 μl 2X SYBR Select Master Mix (Life Technologies, #4472908), 200 nM final concentration of each forward and reverse primer, and 2 μl cDNA in MicroAmp Fast Optical 96-well plates (Life Technologies, # 4346907). Plates were sealed with optical adhesive film (Life Technologies, #4360954) and run in an ABI 7500 real time PCR system using the following program: 50°C for 2 min, 95°C for 2 min, 40 cycles defined as 95°C for 15 sec, 58°C for 15 sec, 72°C for 1 min, followed by a melt curve. Relative expression in the samples of interest was calculated using the ∆∆Ct method [26] for relative quantifications of each gene normalized to GAPDH (Bm5699). The correlation coefficient between Illumina RNA sequencing and qPCR data was analyzed by the Pearson test, with a statistical significance *p*<0.01.

## Results

### Sequencing and mapping

The total number of transcripts identified in each sample and the number of sequence reads for each cDNA library are shown in Table 1. An average of 76.65% of the total number of high quality sequence reads were mapped to the *B*. *malayi* transcriptome after elimination of ambiguous sequence matches. Alignment summary metrics are shown in Table 1. The number of sequence reads mapped per gene varies from one to > 49,000. Between 93.94% and 95.40% of all sequence reads in each library mapped to a transcript.

### Changes over time

We used gene expression levels from the transcriptome of worms immediately after extraction from jirds as the baseline/control and compared gene expression levels in the other three transcriptomes (upon arrival at McGill, after 48 h in culture and after 5 days in culture) against this baseline to identify genes with differential expression in each sample relative to time.

**Fig 2:**
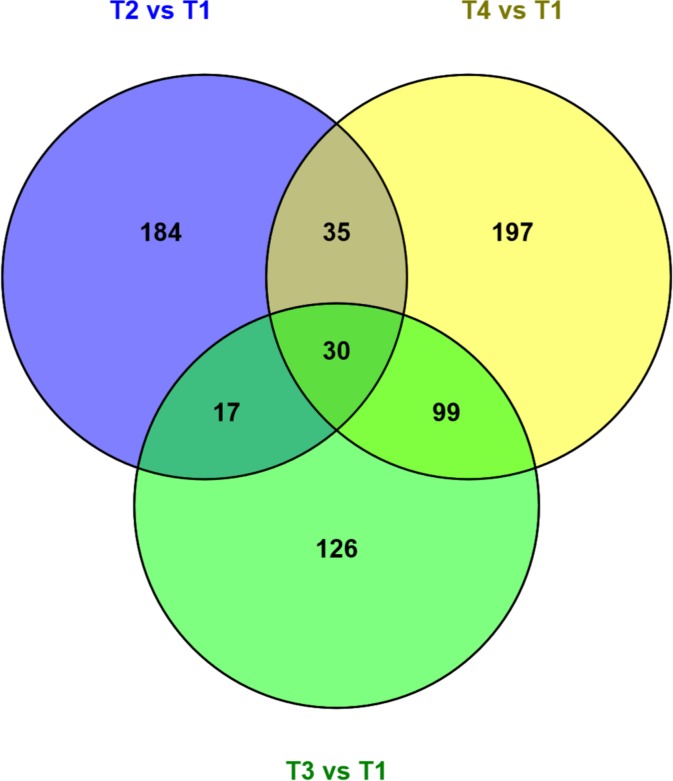
Venn diagram showing the number of overlapping genes across pairwise comparisons of each time point compared to baseline. Venn diagram was created using Venny 2.0.

Pairwise comparisons between time points revealed 138 to 562 differentially expressed genes after applying log_2_ fold-change cutoffs of +1.0 and -1.0 (Tables 2 and S2). Between 35.4 and 47.1% of differentially expressed genes could be assigned GO terms, while between 49.8 and 65.2% had a *C*. *elegans* ortholog. The highest number of differentially expressed genes was observed between T3 and T2, followed by T4 compared to T2. The lowest number of changes resulted from the comparison between T4 and T3. Comparing each time point (T2, T3, and T4) to baseline (T1), we found 30 differentially expressed genes which overlap across all three comparisons. The greatest number of differentially expressed genes in common was 99 across the comparisons T3 vs T1 with T4 vs T1. Among 266 genes dysregulated at T2 compared to T1, 219 returned to baseline levels at T4 (48 h in culture), representing 82% recovery of the initial perturbation. At T4, 35 genes returned to perturbed levels, as was already the case at T2 compared to baseline. Overlaps among the 3 comparisons are displayed in Fig 2. Genes encoding myosin tail family proteins, cadherin domains and proteins orthologous to *C*. *elegans* titin were particularly represented.

**Table 2:** Summary of number and nature of differentially expressed genes over time after removal from host.

	T2 vs T1	T3 vs T2	T3 vs T1	T4 vs T3	T4 vs T2	T4 vs T1
Total DE genes	932	2169	697	491	1252	884
Total DE genes (cutoff log_2_ fold-change ±1.0)	266	562	272	138	412	361
DE genes log_2_ fold-change >1.0	101	320	128	59	294	182
DE genes log_2_ fold-change <-1.0	165	242	144	79	118	179
Sequences with GO terms (cutoff log_2_ fold-change ±1.0)	115	245	118	65	146	138
Sequences with *C*. *elegans* ortholog	151	331	163	90	205	208

A log_2_ fold-change of ±1.0 was applied. T1: upon isolation from host; T2: 24 h after shipping; T3 and T4: after 48 h and 5 days in culture, respectively. GO terms were provided by Nematode.net v 4.0.

Applying the cutoff (log_2_ fold-change ±1.0; i.e., keeping genes with log_2_ fold-change > 1.0 and < -1.0), the proportion of differentially expressed genes represented a maximum of 4.8% of the number of transcripts that were mapped for each time point. Without this cutoff, up to 18.7% of those total transcripts were differentially expressed (in T3 vs T2).

**Fig 3:**
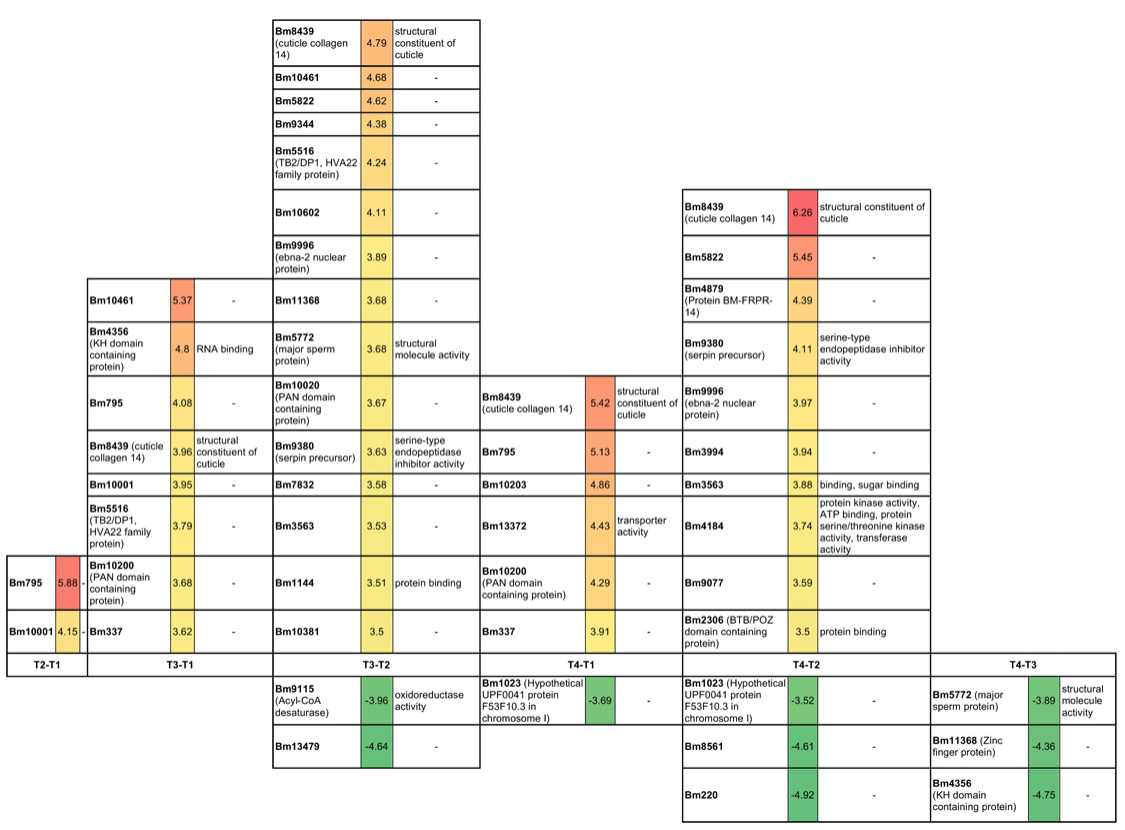
Differentially expressed genes in each time point comparison, with the highest fold-changes. A log_2_ fold-change of +3.5 and -3.5 was applied, respectively. GO terms were retrieved from Nematode.net. Green indicates down-regulation and red, up-regulation.

Fig 3 displays the 32 most prominently differentially expressed genes (log_2_ fold-change cutoff: +/- 3.5). GO annotations (Nematodes.net) show that a collagen-related protein (Bm8439) is among the most profoundly up-regulated genes. Similarly, a serpin precursor (Bm9380) was strongly up-regulated in several comparisons.

**Fig 4:**
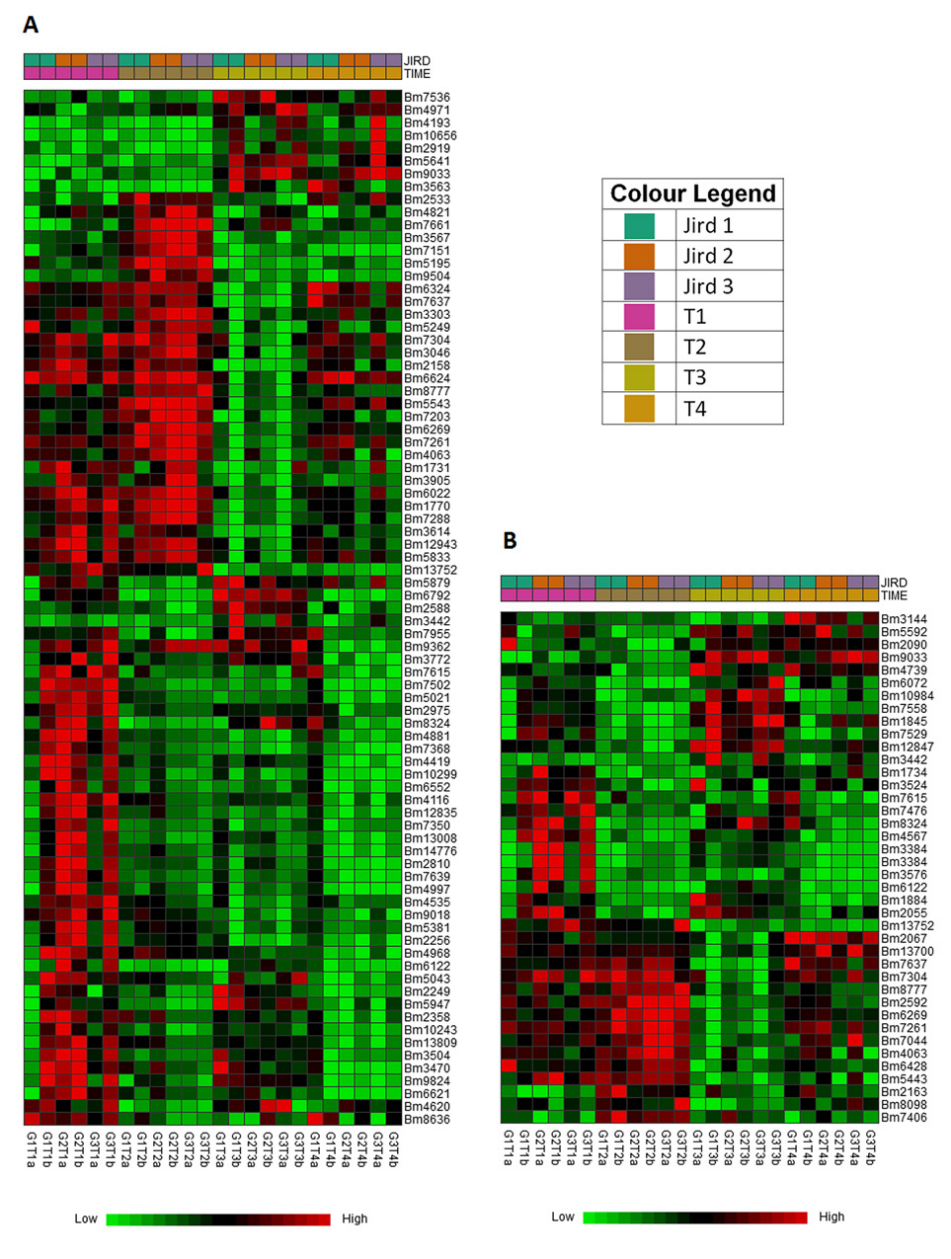
Timewise comparison of gene expression. A. Heat map showing pairwise comparison of genes from GO enrichment analysis. B. Heat map showing pairwise comparison of genes found in enrichment pathway analysis. Genes were normalized using the trimmed mean of M-values normalization method in edgeR and clustered according to Euclidean distance metrics. Green indicates relative down-regulation and red relative up-regulation. Values on the X-axis represent the sample identifications at each time point. Each sample identification is coded by the jird number from which it originated, the time point at which it was analyzed, and the sample replicate letter (a or b).

Comparing T3 to T2, T4 to T2 and T4 to T1 revealed the most important changes, with over 300 differentially expressed genes. Comparing T4 to T3 revealed the lowest number of differentially expressed genes. Statistically significantly enriched GO terms in the T2 vs T1 comparisons were mainly related to regulation of developmental and multicellular organism growth, whereas terms involving phagocytosis and apoptotic cell clearance were most enriched in the T4 vs T2 comparison (S4 Table) and terms related to the nervous system in T3 vs T1, T3 vs T2, and T4 vs T1. Fig 4 shows the timewise expression changes of these genes from GO and pathway enrichment analysis. Below, we present the changes observed comparing T4 to T1 and T4 to other time points in greater detail.

### Transcriptomic profile after 5 days in culture compared to baseline

Among functionally annotated up-regulated genes at T4 compared to T1, a gene encoding a cuticle collagen showed the largest change in expression (log_2_ fold-change = 5.42). A *B*. *malayi* serpin precursor (Bm9380; log_2_ fold-change = 3.1), as well as a gene annotated as Epstein–Barr virus (EBV) nuclear antigen 2 (*ebna-2*; Bm9996; log_2_ fold-change = 3.26), were among the most up-regulated at T4 compared to T1. Bm9996 is also orthologous to a mini-collagen protein (C1IS34, *Malo kingi*), with 75.6% identity (E-value = 14 * 10^−21^).

Genes encoding neuropeptide precursors and neuropeptide receptors were also prominently represented in the up-regulated fraction, including a FMRFamide-like neuropeptide precursor (Bm-flp-11; log_2_ fold-change = 2.87), a neuropeptide receptor (Bm-frprp-14; log_2_ fold-change = 2.59), and a corticotropin-releasing factor receptor precursor (Bm2293; log_2_ fold-change = 2.04) (S2 Table). A dual-specificity phosphatase (Bm12973; log_2_ fold-change = 2.46), and a neurotransmitter transporter with sodium symporter activity (Bm-SNF-11; log_2_ fold-change = 2.90) were also among the genes up-regulated after 5 days in culture. GO term and pathway analysis showed enrichment of terms related to neurogenesis and nervous system development (Table 3) and enrichment of the Wnt and cadherin signaling pathways (S3 Table).

**Table 3:** Distribution of the most enriched biological processes after 5 days in culture compared to baseline (T1).

GO Term	GO Term ID	Input Genes	Reference Genes	P-Value
neuron projection development	GO:0031175	7	161	0.00739
cell projection organization	GO:0030030	7	161	0.00739
neuron differentiation	GO:0030182	7	164	0.008112
neurogenesis	GO:0022008	7	164	0.008112
neuron development	GO:0048666	7	164	0.008112
nervous system development	GO:0007399	7	164	0.008112
generation of neurons	GO:0048699	7	164	0.008112
dendrite development	GO:0016358	4	60	0.010368
neuron recognition	GO:0008038	5	96	0.011260
cell recognition	GO:0008037	5	96	0.011259
axonal fasciculation	GO:0007413	5	96	0.011259
cellular component morphogenesis	GO:0032989	9	274	0.013990
axon development	GO:0061564	6	143	0.014837
protein complex assembly	GO:0006461	7	214	0.028819
protein complex biogenesis	GO:0070271	7	214	0.028819

Analysis was performed using KOBAS 2.0 with the *C*. *elegans* orthologs of differentially expressed genes that met a threshold fold change of +/- 1.0 and FDR<0.01.

Applying a log_2_ fold-change cutoff of -3.5, only one functionally annotated gene had decreased in expression at the end of the experiment (log_2_ fold-change = -3.69), compared to T1. This hypothetical protein, F53F10.3 (Bm1023), is annotated as a probable mitochondrial pyruvate carrier in *C*. *elegans*.

The T4 vs T1 comparison resulted mainly in significantly enriched GO terms related to the nervous system (Table 3).

### Changes between the last two time points

The comparison between T4 and T3 revealed 138 differentially expressed genes. We noted significant up-regulation of Bm8519 (log_2_ fold-change = 3.06). This gene is annotated as pherophorin-dz1 in GenBank, but its function has not been characterized. The sequence contains a ground-like domain which includes a characteristic pattern of conserved cysteine residues. Fifteen collagen genes, as well as the neuropeptide precursor Bm-flp-11, were expressed at higher levels after 5 days in culture (T4) than after 2 days (T3), showing log_2_ fold-changes between 1.0 and 2.3.

GO term enrichment analysis between the last two time points revealed only one statistically significant enriched GO term: inductive cell migration (GO:0040039). This biological process is defined by the « migration of a cell in a multicellular organism that, having changed its location, is required to induce normal properties in one or more cells at its new location ».

### Cuticle collagens

Twenty-five cuticle collagen genes (log_2_ fold-change ± 1.0) were significantly differentially expressed. Ten were down-regulated at T2 and/or T3 compared to T1 (Bm11024, Bm11095, Bm7894, Bm9941, Bm9021, Bm8043, Bm2854, Bm4507, Bm8444, Bm6324). Fifteen were up-regulated at T4 compared to T3 (Bm11024, Bm11095, Bm2854, Bm3144, Bm4507, Bm4605, Bm6324, Bm6324, Bm6421, Bm7408, Bm7894, Bm8043, Bm8439, Bm9021, Bm9092). One, Bm8439, was strongly up-regulated over time (log_2_ fold-change between 1.47 and 6.26), especially between T3 and T4 compared to T1 and T2, but no significant change in its expression was observed between T2 and T1. With the exception of Bm8439, Bm1249, Bm10414, and Bm9504, all collagen genes showing differential expression were down-regulated at T3 and T2 compared to the earlier time points (T2 and T1, respectively), but became more up-regulated over time.

### Other significantly differentially expressed genes

Several serpins were significantly up-regulated upon arrival (Bm1937; log_2_ fold-change = 1.87) and at T3 (Bm1988; log_2_ fold-change = 1.78) compared to T1. Expression levels of Bm1937 decreased by T4, returning to levels observed at extraction from the host. A substantial increase in expression of a serpin precursor occurred over time in culture (T3 and T4) (Bm9380; log_2_ fold-changes between 2.66 and 4.11) compared to T1 and T2.

We observed a very large increase in the expression of Bm3563 at T3 and T4 (log_2_ fold-change = 3.53 at T3 vs T2 and log_2_ fold-change = 3.88 at T4 vs T2). This gene is homologous to lymphocyte antigen 75 in *Ascaris suum* (48% sequence identity, E-value = 140*10^−51^) and clec-1 (C-type lectin) in *C*. *elegans* (37.9% sequence identity, E-value = 5.2*10^−27^).

### Host-related variability

Few genes (0–7) were differentially expressed in worms retrieved from different jirds upon extraction (S5 Table and S1 Fig). Pearson correlation coefficients between samples at T1 were very high (0.954 to 0.995; S6 Table). Six genes were significantly differentially expressed between worms from jird 3 and jird 2 and four genes between jirds 2 and 1. Five of the seven genes were annotated as hypothetical. A ShTK domain-containing protein (Bm7941) was down-regulated in worms extracted from jird 2 and up-regulated in worms from jird 3, and a Ser/Thr protein phosphatase family protein partial mRNA (Bm7394) was significantly up-regulated in jird 3. A three-dimensional Principal Component Analysis graph (S2 Fig) shows the spatial relationship of the level of proximity between gene expression data from biological replicates from each jird. Biological Coefficients of Variation (BCV) [27] were calculated using the EdgeR (V 3.12.0) Bioconductor package in RStudio for pairwise comparisons at T1 and showed the lowest BCV between jird 2 and 3 (S7 Table).

No gene was significantly differentially expressed between jird 3 and jird 1.

### qPCR validation

**Fig 5:**
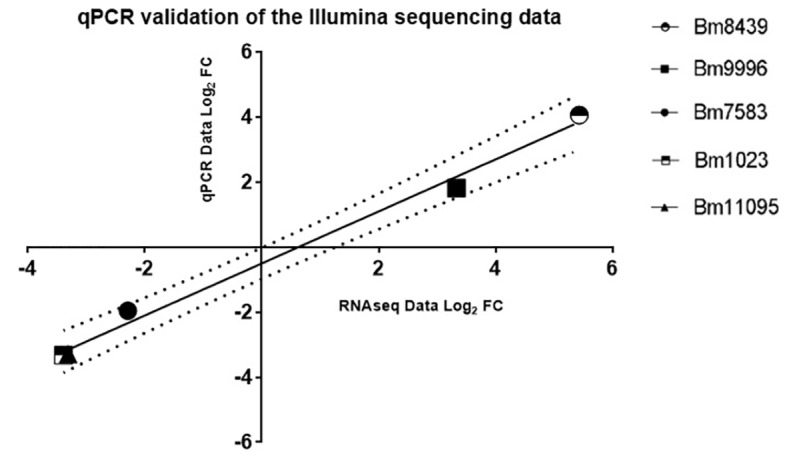
Correlation between RNAseq and qPCR data from 5 genes at different time points. Fold-change values of the selected genes are displayed in S1 Table. The correlation coefficient between RNAseq (x-axis) and qPCR (y-axis) data (log_2_ fold-change) analyzed by the Pearson test was 0.9961 with a statistical significance *p*<0.01.

We performed qPCR to confirm gene expression levels measured by sequencing. We randomly chose 5 genes that were significantly up- or down-regulated at different time points compared to T1. A robust correlation was found between Illumina RNA sequencing and qPCR data, with a correlation coefficient r = 0.9961, analyzed by the Pearson test (*p*<0.01) (see Fig 5 and S1 Table).

## Discussion

We assessed the impact over time of the maintenance of adult *B*. *malayi* females *in vitro* at the transcriptomic level. To ensure robustness of the data, we used worms from three different hosts and processed them in duplicate groups, for a total of six samples for each time point. The strong correlation between RNA sequencing data and qPCR for 5 genes confirm the accuracy of Illumina sequencing and the suitability of the approach for comparative transcriptomic studies. The qPCR results further emphasize the rigor of the study.

Shipment of worms after removal from their hosts triggered a global but transient perturbation of the mRNA profile. Several elements could have contributed to this global perturbation (e.g., shipment in non-supplemented culture media, varying temperatures during this process) which cannot be separately evaluated. Up to 3 worms per group of 8 were immotile and considered dead at the end of the experiment and were excluded from the study. Hence, culturing under these conditions for so long (6 days after removal from hosts) is not optimal, because of the significant loss of viability. We observed a minor loss at T3 (48 h in culture): 3/48 worms considered for extraction at T3 were immotile and excluded. Our analysis revealed up to 562 differentially expressed genes in pairwise comparisons, representing up to 4.84% of all genes. This number stems from the fact that we applied a fairly permissive cutoff (log_2_ fold-change of ± 1.0), and is comparable to the number of genes observed to be differentially expressed under oxidative stress in *C*. *elegans* [28]. Using a much more stringent cutoff at ± 3.5 revealed a maximum of 32 differentially expressed genes in pairwise comparisons. This highlights the very small proportion of strongly dysregulated gene expression over 5 days in culture. Of particular interest are genes encoding cuticle collagens and serpins, which are typically highly expressed in adult females, eggs and embryos compared to other stages [29].

The pattern of gene expression of worms obtained from 3 different jird hosts was in general very highly conserved, with Pearson correlation coefficients ranging from 0.954 to 0.995 between samples.

The nematode cuticle, which is predominantly composed of cross-linked collagens, is a highly impervious barrier between the animal and its environment and is required for the maintenance of body morphology and integrity [30, 31]. It plays a critical role in locomotion via attachments to body wall muscles [32]. Cuticle collagens are believed to be involved in stress resistance, defense against environmental perturbations and longevity in *C*. *elegans* [28]. That we observed twenty-two collagen genes to be substantially and increasingly strongly up-regulated over time in culture may indicate that culture places increasing stress on the worms. Several genes encoding other cuticle collagens were down-regulated at T2 and T3 compared to T1, but up-regulated at T4 compared to earlier time points.

The serpins are a superfamily of serine protease inhibitors that employ a suicide substrate-like mechanism [33]. They are 350–500 amino acids in length and fold into a conserved structure. Serpins have been identified in animals, plants, insects, and certain viruses [34]. At least 14 serpins are predicted in the *Brugia* genome, but only two have been characterized: Bma-SPN-1 and Bma-SPN-2, which are exclusively secretory [35–37]. Previously, Bm9380 (related to Bma-SPN-2) and Bm1988 were found to be highly abundant in the excretory-secretory products of microfilaria [38, 39]. Bma-SPN-2 is exclusively expressed in microfilariae and elicits a strong but short-lived immune response in mice and humans [40]. It may play a role in protection from immunity by inhibiting neutrophil function [35, 41]. Bma-SPN-1, in contrast, is expressed in all life cycle stages, but little has been reported about its target protease(s) [35]. None of the serpin-encoding genes that were dysregulated in the present study mapped to Bm-SPN-1. Interestingly, we found a serpin precursor (Bm9380) to be increasingly up-regulated until the end of the experiment. In *C*. *elegans*, intracellular serpins regulate proteolytic pathways leading to cell death in a pro-survival manner. SRP-6, for example, functions by blocking intestinal cell lysosomal disruption, cytoplasmic proteolysis and death induced by hypotonic shock, thermal stress, oxidative stress, hypoxia, and cation channel hyperactivity [42]. Minutes after hypotonic shock, *srp*-6 null worms undergo a catastrophic series of events resulting in lysosomal disruption, cytoplasmic proteolysis, and death [42]. The up-regulation of serpins Bm9380, Bm1937 and Bm1988 upon arrival suggests a pro-survival function for these genes.

Our results also showed a very large increase in the expression of a lymphocyte antigen 75/clec-1 homolog (Bm3563) at 48 h (T3) and 5 days (T4) in culture. The C-type lectins in *B*. *malayi* have not been well characterized. However, several pathogens exploit lectin receptors to escape intracellular degradation and to suppress the generation of an efficient immune response [43, 44]. The substantial and persistent up-regulation of Bm3563 up to 5 days in culture is in line with suggested roles in the prevention of cellular degradation.

The *B*. *malayi* genome encodes 14 sequences with ground-like domains. Ground-like genes are referred to as hedge-hog (hh)-related genes. Bm3090, which contains a ground-like domain, was down-regulated at T3 compared to T2, and Bm14306 was up-regulated in T4 compared to T2. Bm3090 is homologous to the hh-related gene *grl-4* in *C*. *elegans*, which encodes a protein that is expressed in the pharynx, reproductive system, vulva, larval neurons, and larval rectal epithelium [45]. Several hh-like proteins, including *grl-4*, were up-regulated in *C*. *elegans* in response to oxidative stress [28]. In *B*. *malayi*, *grl-4* is highly expressed in the L_3_ infective larval stage and may play a role in molting [46].

One of the genes with the most marked differential expression pattern across several time points was Bm9996. This gene is annotated as Epstein–Barr virus (EBV) nuclear antigen 2 (*ebna-2*). It is the only *ebna-2* gene sequence found in Nematoda. EBNA-2 is an EBV viral transcription factor which can regulate viral and cellular genes and is associated with Burkitt’s lymphoma and Hodgkin’s disease [47]. Interestingly, EBNA-2 is capable of mimicking notch 1 and, although not related by sequence, they have similar biochemical and functional properties [48]. Notch signaling is critical for cell-to-cell communication, development and metabolism [49]. Notch signaling pathway homolog protein 1 is present in *B*. *malayi*. The ebna-2 gene in *B*. *malayi* has been shown to be preferentially expressed in L_3_ and L_4_ stages [29]. A plausible explanation of the function of EBNA-2 in the context of *in vitro* culture is not readily apparent. Its high degree of sequence identity to mini-collagens is interesting and suggests that it may play a structural function. Mini-collagens are small collagen-like peptides containing long stretches of polyproline and many cysteine residues and are a major component of the inner wall of nematocysts in all species of cnidarians [50].

Among other significantly dysregulated genes were several that encode zinc finger domain-containing proteins of the C2H2 type (Bm1469 and Bm3388) and a DHHC zinc finger domain-containing protein (Bm11360). C2H2 zinc finger domains are the most common DNA-binding motifs in eukaryotic transcription factors and can also bind to RNA and target proteins [51]. The DHHC zinc finger domain-containing protein functions in post-translational modification by attaching palmitate via a thioester linkage mainly to cysteine residues [52].

Several dysregulated genes were annotated as hypothetical (Bm2888, Bm5606). GO analysis of the encoded proteins revealed associations with iron binding, oxygen binding, and oxidoreductase activity; two genes which were significantly down-regulated had calcium ion binding GO terms (Bm4715 and Bm3541).

Neuropeptides are well-known modulators of nematode behavior. The gene encoding the neuropeptide precursor Bm-flp-11 was overexpressed after 5 days in culture (T4), compared to all previous time points. In *C*. *elegans*, FLP-11 peptides inhibit pharyngeal activity [53, 54]. In *A*. *suum*, most FLPs exerted an inhibitory effect on oviposition [55]. In line with the trend of neuropeptide precursors to be overexpressed over time, a gene encoding the neuropeptide receptor Bm-frpr-14 was also up-regulated at T3 and T4 compared to the two earlier time points.

After 5 days in culture (T4), we saw an enrichment of GO terms primarily related to the nervous system. Three genes linked to the cadherin and Wnt signaling pathways (Bm3384, Bm3576, and Bm6122) were strongly down-regulated at T4 vs T1. These 3 genes are orthologs of *fmi-1*, *cdh-4*, and *hmr-1* in *C*. *elegans*, respectively. In *C*. *elegans*, *hmr-1* encodes a neuronal classic cadherin involved in regulation of axon fasciculation, with loss-of-function mutations resulting in the disruption of axonal guidance in a subset of motor neurons [56]. Cadherin FMI-1 is mainly expressed in the nervous system in *C*. *elegans* and regulates GABAergic neuronal development. Loss-of-function mutants show patterning defects in the GABAergic ventral D-type (VD) neurons and *fmi-1* mutants show defective axon pathfinding as well as reduced synapse number, aberrant size and morphology [57]. Interestingly, cadherin-4 functions in the same pathway as FMI-1 in the regulation of GABAergic neuron development and plays a role in axon guidance [58, 59]. As the cadherin signaling pathway also converges with the Wnt signaling pathway, it is not surprising that we see an enrichment of both [56]. The significant down-regulation of these genes at T4 is suggestive of nervous system degeneration and a decline in axon regeneration in an aging cellular environment [60, 61].

In the T4 vs T3 comparison, the only GO term found to be significantly enriched, with 4 *C*. *elegans* orthologs (*cocg-1*, *emb-9*, *ccdc-55*, and *let-2)* of Bm6792, Bm7637, Bm2249, and Bm3144, respectively, was “inductive cell migration” (GO:0040039). Interestingly, these genes also have GO terms associated with embryo and larval development, suggesting a role in reproduction. COGC-1 (orthologous to Bm6792 which was down-regulated in T4 vs T3) is required for normal gonadal distal tip cell migration, as well as normal vulval morphology in *C*. *elegans* [62, 63]. Bm2249 (also significantly down-regulated) is orthologous to *ccdc-55* which in *C*. *elegans* also plays a role in distal tip cell migration and larval development [64]. The remaining two genes (Bm7637 and Bm3144) were up-regulated in this comparison and are orthologous to *emb-9* and *let-2*, which encode collagen alpha-1 (IV) chain and collagen alpha-2 (IV) chain, respectively. Type IV collagen is a major component of basement membranes. Mutations in *emb-9* and *let-2* in *C*. *elegans* cause embryonic development arrest [65], suggesting the importance of these genes in embryogenesis. The dysregulation of these 4 genes at T4 vs T3 suggests that reproduction and embryogenesis may be affected after 5 days in culture due to events similar to aging. The enrichment of GO terms at T4 vs T2 related to apoptotic cell clearance and phagocytosis is also consistent with the hypothesis that the animal is degenerating and dying cells are being removed and may suggest that the animal is under oxidative stress, which is known to be a mediator of apoptosis and neuronal cell death [66–68].

We observed differentially expressed genes paralleling oxidative stress responses in *C*. *elegans* (overexpression of collagens, hedgehog proteins, etc.). However, heat shock proteins and ATPases, strongly represented in stressed *C*. *elegans* [28], were not prominent in cultured *B*. *malayi* females (with 1 gene and 8 genes respectively, in our dataset). In addition, GO terms associated with oxidative stress in *C*. *elegans* [28] did not overlap substantially with our findings. In *C*. *elegans*, *elt-2* and *osm-12* are markers of osmotic stress [69] and in the present study we saw the up-regulation of Bm2533 (orthologous to *elt-2*) upon receipt (T2), which was down-regulated after 48 h in culture, and the up-regulation of Bm7137 at 48 h which returned to baseline levels by 5 days. This gene is annotated as a hypothetical gene in *B*. *malayi* yet is orthologous to *osm-12*. In hyperosmotic stress conditions in *C*. *elegans*, glycerol-3-phosphate dehydrogenase is highly up-regulated [70]. We did not detect dysregulated expression of *gpdh-1*.

Finally, the variability attributable to the host was only minor. The number of significantly differentially expressed genes was low (0–7 genes with no fold-change cut-off) and the high values obtained from the Pearson correlation of coefficients between samples documents the similarity of sample gene sets immediately after worm extraction. The biological coefficient of variation between replicates ranged from 14.4 to 25.3%. Moreover, the jirds used to maintain *B*. *malayi* are an outbred strain, paralleling human populations.

In summary, we characterized the transcriptomic effects of *in vitro* maintenance of adult *B*. *malayi* females after up to 5 days in culture, i.e. 6 days after isolation from a host. We suggest that environmental changes encountered after removal from the host and shipping provoke important perturbations in gene expression. We noted changes in expression levels of a few genes that are general indicators of stress, best illustrated by strong increases in expression of genes encoding cuticle collagens. We conclude that the *in vitro* culture system is a valuable study tool after the worms are allowed to acclimatize to a new environment and suggest that the stress of removal and shipping can be partially overcome after 48 h in culture. *In vitro* cultivation, however, was not free of stress for the worms, as new dysregulated genes appeared at every time point. We suggest that culture of this parasite under the conditions used here (no feeder cell layer) should not be extended past 6 days post removal from hosts. After that, more worms are expected to die, highlighting the need for rigorous controls.

## Supporting Information

S1 Fig Focus view heatmap showing the pairwise gene expression comparisons between duplicate groups of pooled worms upon extraction (T1) from 3 different jirds. The heatmap was created using the TMM-Normalized expression matrix from edgeR in networkanalyst.ca.
(TIF)

S2 Fig Principal Component Analysis (PCA) graph showing the relationship between gene expression datasets among the duplicate samples from 3 jirds upon extraction (T1). (TIF)

S1 Table qPCR results validation. Five genes were chosen for validation. The primers used, the qPCR amplification efficiencies, and the correlation coefficient of determination of the slope of the standard curve for each gene are given.
(DOCX)

S2 Table Timewise comparisons of differentially expressed genes. All genes with a log_2_ fold-change < -1.0 or > +1.0 are listed for all comparisons between time points.
(XLSX)

S3 Table Pathway enrichment analysis. All enriched pathways (p<0.05) analyzed using KOBAS 2.0 for each pairwise comparisons.
(DOCX)

S4 Table Gene Ontology Enrichment tables for other timewise comparisons. Tables containing top enriched GO terms for T2 vs T1 (A), T3 vs T1 (B), T3 vs T2 (C), and T4 vs T2 (D). T4 vs T3 is not displayed, as only “inductive cell migration” was found to be significantly enriched.
(DOCX)

S5 Table Host related variability. All genes with a log_2_ fold-change < -1.0 or > +1.0 are listed for all comparisons between biological replicates coming from three different hosts.
(XLS)

S6 Table Proximity matrix of Pearson correlation coefficients between variables upon extraction of worms from hosts (T1). The matrix was generated using XLStat and shows the similarity coefficients between all gene expression variables generated at T1.
(DOCX)

S7 Table Common dispersion values and biological coefficients of variation for jird pairwise comparisons upon extraction from hosts (T1). Common dispersion values and biological coefficients of variations were calculated for each pairwise comparisons between jirds using the EdgeR (V 3.12.0) Bioconductor package in RStudio.
(DOCX)
