# Supplementary figures and images for: The Effect of In Vitro Cultivation on the Transcriptome of Adult Brugia malayi
Source: PLoS Negl Trop Dis. 2016 Jan 4;10(1):e0004311. doi: 10.1371/journal.pntd.0004311 (PMC4699822; doi:10.1371/journal.pntd.0004311)

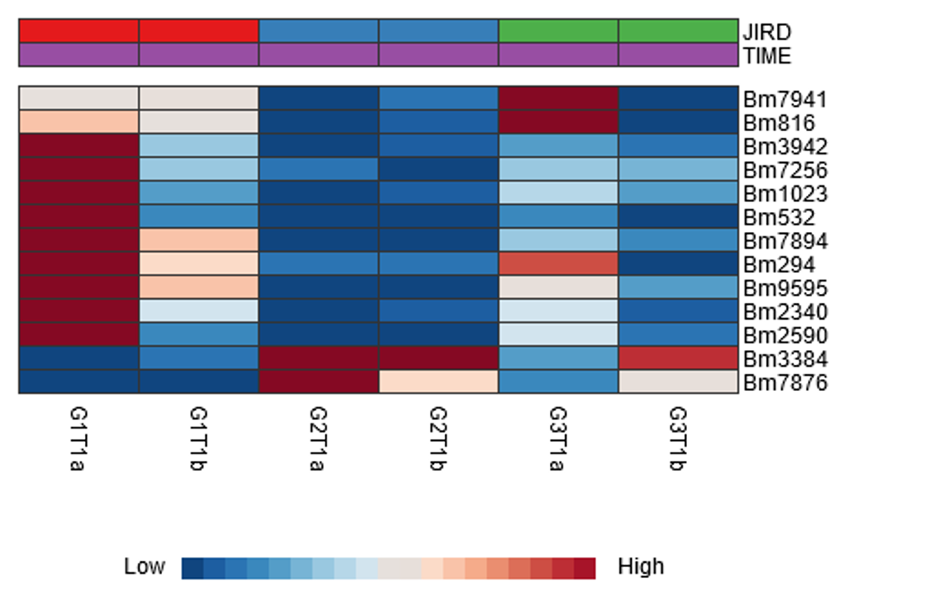

Supplement: S1 Fig — The heatmap was created using the TMM-Normalized expression matrix from edgeR in networkanalyst.ca. (TIF) [file pntd.0004311.s001.tif]

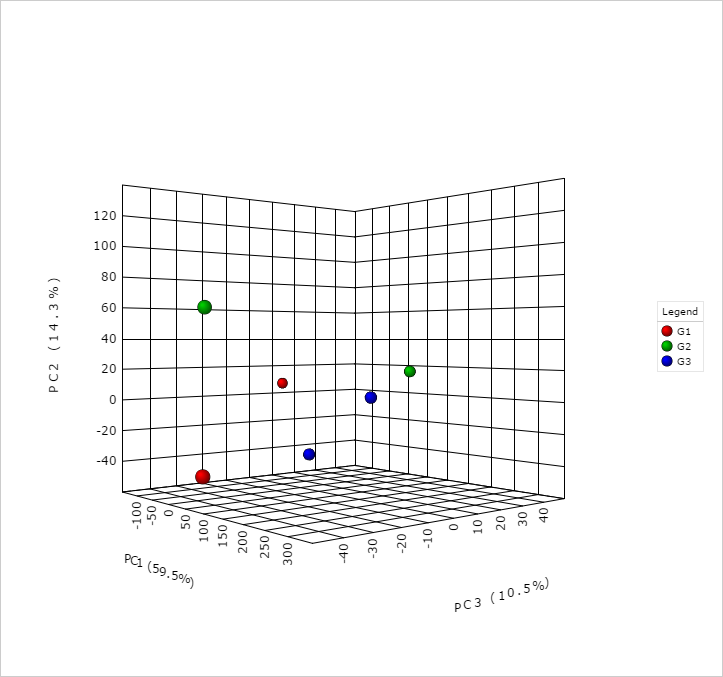

Supplement: S2 Fig — (TIF) [file pntd.0004311.s002.tif]
