# Supplementary material for: The Effect of In Vitro Cultivation on the Transcriptome of Adult Brugia malayi
Source: PLoS Negl Trop Dis. 2016 Jan 4;10(1):e0004311. doi: 10.1371/journal.pntd.0004311 (PMC4699822; doi:10.1371/journal.pntd.0004311)
Supplement: S1 Table — Five genes were chosen for validation. The primers used, the qPCR amplification efficiencies, and the correlation coefficient of determination of the slope of the standard curve for each gene are given. (DOCX) [file pntd.0004311.s003.docx]

**Table S1. Selected candidate reference genes, primers and different parameters derived from qRT-PCR analysis.**

| Gene name | Bm ID | Primer sequences  (forward/reverse) | Amplicon  length (bp) | Amplification  efficiency (%) | *R^2^ |
| --- | --- | --- | --- | --- | --- |
| GAPDH | Bm5699 | TTTCTGCAGAGGGAGGCAAG  TCAGCGGGATCTTTGCTGTT | 85 | 91.09 | 98.6 |
| Cuticle collagen 14 | Bm8439 | GCACCAGGGCCAATTGG  TTGGGCAATGATCACAACCA | 55 | 103.48 | 97.3 |
| EBNA-2 nuclear protein | Bm9996 | CACCACCCGCACCTTACG  CCAAAACCATAGCCGACTCCTA | 58 | 96.01 | 99.6 |
| Tubulin alpha-2 chain | Bm7583 | TGCTGCAATTTCCATGGTTAAA  TCGGGCACCAATCAACAAA | 60 | 92.16 | 88.9 |
| Cuticle collagen C09G5.5 | Bm11095 | ATCGCATTTTTGTTTGGTTCAA  GAAGAGCACATGCACAGATAGCA | 64 | 82.54 | 99.4 |
| Hypothetical UPF0041 protein F53F10.3 | Bm1023 | TTGGGGACCAACGATAAAAT  GCTGTATCGTGTCCAAATGC | 130 | 78.13 | 87.8 |

*R^2^, coefficient of determination of the slope of the standard curve
