# Supplementary material for: The Effect of In Vitro Cultivation on the Transcriptome of Adult Brugia malayi
Source: PLoS Negl Trop Dis. 2016 Jan 4;10(1):e0004311. doi: 10.1371/journal.pntd.0004311 (PMC4699822; doi:10.1371/journal.pntd.0004311)
Supplement: S3 Table — All enriched pathways (p<0.05) analyzed using KOBAS 2.0 for each pairwise comparisons. (DOCX) [file pntd.0004311.s005.docx]

**Table S3.** **Distribution of the top enriched pathways (p<0.05) for each pairwise comparison. Enrichment analysis was performed using KOBAS 2.0.**

| Comparison | Enriched Pathway | Input Genes | Reference Genes | P-Value |
| --- | --- | --- | --- | --- |
| T2 vs T1 | Non-homologous end-joining | 2 | 6 | 0.002526 |
|  | ABC transporters | 2 | 20 | 0.019099 |
|  | Metabotropic glutamate receptor group III pathway | 2 | 12 | 0.025691 |
|  | Nicotinic acetylcholine receptor signaling pathway | 4 | 62 | 0.028822 |
|  | N-Glycan biosynthesis | 2 | 34 | 0.047775 |
| T3 vs T1 | Huntington disease | 3 | 34 | 0.017947 |
|  | MAPK signaling pathway | 3 | 106 | 0.054171 |
| T4 vs T1 | Cadherin signaling pathway | 3 | 21 | 0.002393 |
|  | Wnt signaling pathway | 3 | 70 | 0.052000 |
| T3 vs T2 | Nitrogen metabolism | 3 | 13 | 0.002540 |
|  | Glutamine glutamate conversion | 2 | 5 | 0.004653 |
|  | Wnt signaling pathway | 5 | 70 | 0.010192 |
|  | RNA degradation | 4 | 54 | 0.018901 |
| T4 vs T2 | Glutamine glutamate conversion | 2 | 5 | 0.001002 |
|  | Nitrogen metabolism | 2 | 13 | 0.010095 |
|  | Pantothenate and CoA biosynthesis | 2 | 15 | 0.012904 |
| T4 vs T3 | Fanconi anemia pathway | 2 | 27 | 0.001276 |
|  | Integrin signalling pathway | 2 | 25 | 0.001816 |
|  | Spliceosome | 2 | 130 | 0.024485 |
|  | Nicotinate and nicotinamide metabolism | 1 | 13 | 0.026228 |
