# Supplementary material for: The Effect of In Vitro Cultivation on the Transcriptome of Adult Brugia malayi
Source: PLoS Negl Trop Dis. 2016 Jan 4;10(1):e0004311. doi: 10.1371/journal.pntd.0004311 (PMC4699822; doi:10.1371/journal.pntd.0004311)
Supplement: S4 Table — Tables containing top enriched GO terms for T2 vs T1 (A), T3 vs T1 (B), T3 vs T2 (C), and T4 vs T2 (D). T4 vs T3 is not displayed, as only “inductive cell migration” was found to be significantly enriched. (DOCX) [file pntd.0004311.s006.docx]

**Table S4a.** **Distribution of the top 10 enriched biological processes after receipt of worms at McGill University (T2) compared to baseline (T1).**

| GO Term | GO Term ID | Number Input Genes | Number Reference Genes | P-Value |
| --- | --- | --- | --- | --- |
| growth | GO:0040007 | 9 | 632 | 0.024489 |
| positive regulation of growth | GO:0045927 | 7 | 444 | 0.028025 |
| positive regulation of developmental growth | GO:0048639 | 7 | 444 | 0.028025 |
| regulation of growth | GO:0040008 | 7 | 444 | 0.028025 |
| regulation of multicellular organism growth | GO:0040014 | 7 | 444 | 0.028025 |
| regulation of developmental growth | GO:0048638 | 7 | 444 | 0.028025 |
| positive regulation of multicellular organism growth | GO:0040018 | 7 | 444 | 0.028025 |
| multicellular organism growth | GO:0035264 | 7 | 444 | 0.028025 |
| developmental growth | GO:0048589 | 7 | 455 | 0.031320 |
| positive regulation of developmental process | GO:0051094 | 7 | 489 | 0.043110 |

**Table S4b.** **Distribution of the top 10 enriched biological processes 48 h in culture (T3) after receipt compared to baseline (T1).**

| GO Term | GO Term ID | Input Genes | Reference Genes | P-Value |
| --- | --- | --- | --- | --- |
| axon development | GO:0061564 | 6 | 143 | 0.009256 |
| ovulation | GO:0030728 | 3 | 35 | 0.011231 |
| macromolecule catabolic process | GO:0009057 | 7 | 202 | 0.013312 |
| protein catabolic process | GO:0030163 | 7 | 202 | 0.013312 |
| organic substance catabolic process | GO:1901575 | 7 | 202 | 0.013312 |
| cellular component organization or biogenesis | GO:0071840 | 19 | 891 | 0.014747 |
| cellular component organization | GO:0016043 | 19 | 891 | 0.014747 |
| neuron projection development | GO:0031175 | 6 | 161 | 0.015537 |
| cell projection organization | GO:0030030 | 6 | 161 | 0.015537 |
| neurogenesis | GO:0022008 | 6 | 164 | 0.016814 |

**Table S4c.** **Distribution of the top 10 enriched biological processes after 48 h in culture (T3) compared to receipt at McGill University (T2).**

| GO Term | GO Term ID | Input Genes | Reference Genes | P-Value |
| --- | --- | --- | --- | --- |
| neurogenesis | GO:0022008 | 12 | 164 | 0.002326 |
| neuron differentiation | GO:0030182 | 12 | 164 | 0.002326 |
| neuron development | GO:0048666 | 12 | 164 | 0.002326 |
| nervous system development | GO:0007399 | 12 | 164 | 0.002326 |
| generation of neurons | GO:0048699 | 12 | 164 | 0.002326 |
| growth | GO:0040007 | 30 | 632 | 0.002748 |
| positive regulation of biological process | GO:0048518 | 26 | 542 | 0.004424 |
| neuron projection development | GO:0031175 | 11 | 161 | 0.005598 |
| cell projection organization | GO:0030030 | 11 | 161 | 0.005598 |
| positive regulation of growth | GO:0045927 | 22 | 444 | 0.005902 |

**Table S4d.** **Distribution of the top 10 enriched biological processes after 5 d in culture (T4) compared to receipt at McGill University (T2).**

| GO Term | GO Term ID | Input Genes | Reference Genes | P-Value |
| --- | --- | --- | --- | --- |
| growth | GO:0040007 | 10 | 632 | 0.029380 |
| locomotion | GO:0040011 | 23 | 1911 | 0.029846 |
| phagocytosis | GO:0006909 | 3 | 89 | 0.034710 |
| membrane organization | GO:0061024 | 3 | 89 | 0.034710 |
| phagocytosis, engulfment | GO:0006911 | 3 | 89 | 0.034710 |
| engulfment of apoptotic cell | GO:0043652 | 3 | 89 | 0.034710 |
| apoptotic cell clearance | GO:0043277 | 3 | 89 | 0.034710 |
| membrane invagination | GO:0010324 | 3 | 89 | 0.034710 |
| positive regulation of growth | GO:0045927 | 7 | 444 | 0.062796 |
| regulation of multicellular organism growth | GO:0040014 | 7 | 444 | 0.062796 |
