# Supplementary material for: The Effect of In Vitro Cultivation on the Transcriptome of Adult Brugia malayi
Source: PLoS Negl Trop Dis. 2016 Jan 4;10(1):e0004311. doi: 10.1371/journal.pntd.0004311 (PMC4699822; doi:10.1371/journal.pntd.0004311)
Supplement: S6 Table — The matrix was generated using XLStat and shows the similarity coefficients between all gene expression variables generated at T1. (DOCX) [file pntd.0004311.s008.docx]

**Table S6: Proximity matrix of Pearson correlation coefficients between variables upon extraction of worms from hosts (T1).**

| Variable | G1T1a | G1T1b | G2T1a | G2T1b | G3T1a | G3T1b |
| --- | --- | --- | --- | --- | --- | --- |
| G1T1a | 1 | 0.981 | 0.954 | 0.964 | 0.988 | 0.977 |
| G1T1b | 0.981 | 1 | 0.982 | 0.988 | 0.993 | 0.995 |
| G2T1a | 0.954 | 0.982 | 1 | 0.991 | 0.973 | 0.985 |
| G2T1b | 0.964 | 0.988 | 0.991 | 1 | 0.980 | 0.994 |
| G3T1a | 0.988 | 0.993 | 0.973 | 0.980 | 1 | 0.989 |
| G3T1b | 0.977 | 0.995 | 0.985 | 0.994 | 0.989 | 1 |
