# Supplementary material for: The Effect of In Vitro Cultivation on the Transcriptome of Adult Brugia malayi
Source: PLoS Negl Trop Dis. 2016 Jan 4;10(1):e0004311. doi: 10.1371/journal.pntd.0004311 (PMC4699822; doi:10.1371/journal.pntd.0004311)
Supplement: S7 Table — Common dispersion values and biological coefficients of variations were calculated for each pairwise comparisons between jirds using the EdgeR (V 3.12.0) Bioconductor package in RStudio. (DOCX) [file pntd.0004311.s009.docx]

**Table S7: Common dispersion values and biological coefficients of variation for gerbil pairwise comparisons upon extraction from hosts (T1).**

| Pairwise Comparison | Common Dispersion Value | Biological Coefficient of Variation (BCV) |
| --- | --- | --- |
| Gerbil 2 vs Gerbil 1 | 0.04752116 | 0.2179935 |
| Gerbil 3 vs Gerbil 1 | 0.06417979 | 0.2533373 |
| Gerbil 3 vs Gerbil 2 | 0.02087929 | 0.1444967 |
